# Enhancing the Recovery of Antioxidant Compounds from Microalgae-Cyanobacteria Consortia Through Alcalase Hydrolysis: A Focus on Bioactive Peptides

**DOI:** 10.3390/md24050184

**Published:** 2026-05-20

**Authors:** Blanca Pardo de Donlebún, Rocío del Álamo, Pilar Águila-Carricondo, Juan Pablo de la Roche, Pilar Gómez-Cortés, Blanca Hernández-Ledesma

**Affiliations:** 1 Department of Bioactivity and Food Analysis, Institute of Food Science Research (CIAL, CSIC-UAM, CEI UAM+CSIC), Nicolás Cabrera 9, 28049 Madrid, Spain; blanca.pardo@csic.es (B.P.d.D.); rocio.alamob@gmail.com (R.d.Á.); 2 Department of Nutrition and Food Science, Faculty of Pharmacy, Complutense University of Madrid (UCM), Plaza Ramón y Cajal s/n, 28040 Madrid, Spain; 3 Microalgae Solutions S.L, Factoría Industrial de Vicálvaro, Nave 5, 28052 Madrid, Spain; paguila@microalgaesolutions.com (P.Á.-C.); jproche@microalgaesolutions.com (J.P.d.l.R.)

**Keywords:** microalgae, cyanobacteria, consortia, protein extraction, bioactive peptides, phenolic compounds, antioxidant capacity

## Abstract

Microalgae and cyanobacteria represent an emerging and sustainable source of bioactive compounds for the food, cosmeceutical, and pharmaceutical sectors. In this study, the potential of two microalgae-cyanobacteria consortia, consortium 1 (C1) consisting of *Chlorella vulgaris* and *Arthrospira platensis*, and consortium 2 (C2) consisting of *Kamptonema* sp., *Nannochloropsis oculata*, *Tetraselmis suecica*, and *Chlorella vulgaris*, as a source of bioactive peptides was evaluated. Firstly, protein extraction from both biomasses was optimized by testing different protein solubilization and precipitation pHs, with pH 10 and pH 5 providing the best results in terms of protein recovery in both cases. Selected protein extracts, with protein contents of 28.50 ± 2.69% (C1) and 8.46 ± 0.45% (C2), were further hydrolyzed with Alcalase, evaluating the impact of the incubation time on peptide release and the antioxidant capacity of hydrolysates. A total of 1 h of hydrolysis proved to be enough for antioxidant capacity increase. In addition, in silico hydrolysis of the proteins identified with Alcalase in C1 and C2 (data are available via ProteomeXchange with identifier PXD077201 and PXD077149 for C1 and C2, respectively) was evaluated, assessing the potential bioactivity of the peptides produced, more specifically their antioxidant capacity. Our findings demonstrate that both microalgae-cyanobacteria consortia are valuable sources of bioactive compounds with antioxidant capacity, with potential interest as functional ingredients for the food, cosmeceutical, and pharmaceutical industries.

## 1. Introduction

Microalgae are unicellular photosynthetic microorganisms that produce a large range of biomolecules, such as proteins, lipids, carbohydrates, and nucleic acids, as well as vitamins and minerals, with variable content depending on the species and physiological responses to biotic and abiotic factors in the surrounding environment [1]. Cyanobacteria are photosynthetic bacteria [2] that can be found in symbiosis with microalgae, resulting in the establishment of microalgae-cyanobacteria consortia. These interactions hold significant interest for biotechnology applications related to human health due to their large production of high-value bioactive metabolites [3,4]. Their low environmental impact production is a key advantage, positioning them as a sustainable solution for meeting the food demands of a growing global population while supporting economic development [5]. Due to their high nutrient content and bioactivity, the use of different species, such as *Dunaliella salina*, *Chlorella vulgaris* or *Tetraselmis chuii*, has been approved in some countries for food, nutraceutical or pharmaceutical purposes [5]. Examples of the main metabolites that can be found in microalgae and whose bioactivity has been demonstrated are bioactive peptides (BAPs), omega-3 and omega-6 polyunsaturated fatty acids (PUFAs), phytosterols, phycobiliproteins, phenolic compounds, carotenoids, and chlorophylls [6]. These compounds have been characterized as exerting antioxidant, anti-inflammatory, antimicrobial, and anticancer activities, among others [1].

Microalgae and cyanobacteria constitute a high-quality and sustainable source of protein, frequently containing protein at levels comparable to or greater than conventional crops. These microorganisms address the challenge of scaling protein production sustainably, thanks to their fast growth cycles and CO_2_ capture, without competing for arable land [7]. In order to use their proteins in food and other products, they are often extracted to obtain high-protein concentrates. Protein solubilization can be achieved through physical (i.e., ultrasound, permeabilization, or freezing), chemical (i.e., alkaline conditions), or enzymatic methods, with an increase in extraction yields observed when these methods are combined [8,9,10,11,12,13]. Subsequently, acidification methods have been usually applied for protein precipitation [11,14].

In addition to their nutritional value and functional properties, these proteins have been described as a source of BAPs. These molecules are defined as fragments of 2–20 amino acids that, after being released from the precursor protein, can trigger positive health effects [15,16]. Multiple studies have identified BAPs from food proteins with different biological activities, such as antioxidant, anti-inflammatory, antimicrobial, antidiabetic, antihypertensive, anticancer, and immunomodulatory activity [17]. The obtention of BAPs can be achieved after hydrolysis processes, gastrointestinal digestion and/or during food processing. In the case of hydrolysis, different methods have been used for BAPs release, with chemical hydrolysis, microbiological fermentation and enzymatic hydrolysis being the most common [18,19]. Enzymatic hydrolysis is the preferred strategy for peptide production in food and pharmaceutical applications due to its clean process, which eliminates the chemical waste and hazardous substances associated with chemical hydrolysis [20]. Several commercial enzymes, such as trypsin, papain, pepsin, bromelain, Alcalase or chymotrypsin have been widely used [16]. The selected enzyme will determine final aspects such as the degree of hydrolysis (DH), the amino acid sequence of the released peptides and the potential bioactivity [21]. Among enzymes, Alcalase has been considered one of the most effective because of its capacity to produce hydrolysates with high DH [21]. Mahdieh et al. [12] used this enzyme to obtain protein hydrolysates from *Arthrospira platensis* (*A. platensis*), isolating the tripeptide IQP with demonstrated antihypertensive and antioxidant properties. Nevertheless, the number of studies on the effects of microalgae BAPs is still limited [22], and so far, there are no studies focused on the potential of microalgae-cyanobacteria consortia as a source of BAPs. Therefore, this study employed an integrated in silico and in vitro framework to comprehensively map the full proteome of two distinct microalgae-cyanobacteria consortia, with the goal of extracting and identifying high-value proteins as precursors for BAPs.

## 2. Results and Discussion

### 2.1. Characterization of Microalgae-Cyanobacteria Consortia Biomasses

Protein, carbohydrate, lipid, moisture, and inorganic material contents of the two microalgae-cyanobacteria consortia (C1 and C2) biomasses are shown in Table 1. Results are expressed as % dry weight (dw).

Regarding the protein content, the values obtained by both methods (Kjeldahl and BCA assays) were similar, indicating that the conversion factor (5.95) used in the Kjeldahl method was suitable to convert the nitrogen content into protein. The protein content of C1 was around 50%, in accordance with the values reported in the literature for each of the species that composed this consortium, being 50% for *Chlorella vulgaris* (*C. vulgaris*) [23,24] and between 53 and 70% for *A. platensis* [23,25]. C2 was composed of *Kamptonema* sp., *Nannochloropsis oculata* (*N. oculata)*, *Tetraselmis suecica* (*T. suecica*), and *C. vulgaris*. The protein content determined by both assays was around 25%, generally lower than the values reported for the individual microalgae and cyanobacteria species [23,24,26,27,28,29,30].

The lipid content of C1 and C2 was low, 6.22 ± 1.37 and 2.71 ± 0.72%, respectively. In this regard, there is a high variability reported in the literature for the lipid content of these microorganisms, which can be due to the cultivation conditions used [31]. With regard to carbohydrate content, C1 presented a value of 19.35 ± 0.74%, which was consistent with values reported in the literature for the individual species contained in this consortium [23,25], and C2 presented a carbohydrate content of 35.48 ± 0.81%. Apart from that, both types of biomasses had a moisture content of approximately 5%. Furthermore, in terms of inorganic matter content, a higher value was observed in C2 (37.07 ± 0.08%) that should be attributed to the use of seawater for the cultivation of this specific biomass. The variation observed in our composition results compared to previous studies might be due to the different contribution of each microalga and cyanobacterium species to the complete biomass, the type of strains, or the culture conditions [3,5,31,32].

### 2.2. Identification of the Proteins Present in the Microalgae-Cyanobacteria Consortia Biomasses

After carrying out the in-gel digestion of the proteins and the RP-LC-MS/MS analysis, 9389 and 7152 total peptide spectrum matches (PSMs) were identified in C1 and C2, respectively, using the PEAKS Studio v11.5 search engine and the module SPIDER [33]. Detailed information on the proteins, such as the corresponding accession numbers, PEAKS protein scores (−10lgP), coverage (%), peptide counts (total and unique), average mass, description, and types of peptide modifications (PTMs) of the proteins detected with ≥2 unique peptides/protein group, are compiled in the Supplementary Table S1 (C1) and Table S2 (C2). These results included 9186 and 7031 PSMs from MS/MS scans (MS/MS scans that are associated with a specific peptide spectrum), 4738 and 5567 features (identified by database search only), 4584 and 5438 peptide sequences with modifications (without isoleucine (I)/leucine (L) differentiation), and 3831 and 4289 peptide sequences without modifications (with I/L differentiation), for C1 and C2, respectively.

PEAKS organized proteins into groups that included all those identified from the same set of peptides [34]. In this work, 1428 (1315 proteins from *A. platensis* and 113 from *C. vulgaris*) and 866 (649 proteins from *Kamptonema* sp., 155 from *C. vulgaris*, 55 from *N. oculata*, and seven from *T. suecica*) proteins were identified. Of all of them, 878 and 554 proteins were identified using two or more unique peptides, and 574 and 756 correspond to groups of proteins for C1 and C2, respectively.

Guadalupi et al. [35] evaluated the effect of different protein extraction methods on the proteome of *C. vulgaris* biomass, identifying up to 771 proteins. From the most abundant proteins identified by these authors, glyceraldehyde-3-phosphate dehydrogenase, ATP synthase subunits alpha and beta, Tr-type G domain-containing protein, and cytochrome b559 subunit alpha were also identified in our study. Moreover, in the work of Bianco et al., [36] the proteome of *C. vulgaris* was linked to the possible allergenicity of the proteins. They could identify a total of 499 proteins from this microalga, but only 26 of them had more than 10% of coverage. Of these, some of them were also identified in our C1 biomass, such as photosystem II D2 protein, chlorophyll a-b binding protein and fructose-bisphosphate aldolase. In the article published by Ismaiel et al., [37] the variation in the expression of *A. platensis* proteins under stressful growth conditions (pH, salinity, presence of metals, etc.) was evaluated. Some of the proteins identified coincided with those identified in our work, such as cell division protein FtsZ (growth function), adenosylhomocysteinase (regulation function), ribulose-bisphosphate carboxylase (carboxylase function), fructose-1,6-bisphosphatase (carbohydrate metabolism), glucosylglycerol-phosphate synthase, and phosphoribulokinase (transfer function). On the other hand, Ji et al. [38] reported 593 proteins in *A. platensis*, with 155 overlapping our identified proteome.

Regarding the most abundant microorganism in C2, *Kamptonema* sp., scarce information about its proteome can be found in the literature. Shishido et al. [39] analyzed the genome and protein sequences of this cyanobacterium, highlighting some proteins described in the Kyoto Encyclopedia of Genes and Genomes (KEGG). Among these, ferredoxin-nitrite reductase was the sole protein detected in our C2 biomass. Our work provides new information about the proteome of *Kamptonema* sp., after identifying a total of 649 proteins. Regarding the proteome of *N. oculata*, in the work of Tran et al., [40] the change in protein expression in the microalga growing under nitrogen-deplete conditions was evaluated. These authors identified 1487 total proteins, from which ribosomal proteins and ATP synthase were also identified in C2. Also, Hamzelou et al. [41] investigated the potential allergenicity of *N. oculata* proteins after their extraction by non-food grade (NFG) and food grade (FG) methods, identifying 1373 and 464 proteins, respectively. These results highlighted the influence of extraction methodology on the protein profile obtained from this microalga. From the FG extracted proteins, 31 were described as potentially allergenic and of them, glyceraldehyde-3-phosphate dehydrogenase and chaperone protein DnaK were also identified in our study. Consequently, this finding emphasizes that the safety assessment of alternative protein sources, including microalgae, must include a rigorous evaluation of their allergenic potential. Guzman et al. [42] focused on the identification of antimicrobial peptides produced by *T. suecica*. They searched in the National Center for Biotechnology Information (NCBI) database for sequences that matched with protein regions identified in this species. Several peptides were also found in proteins identified in our biomass, such as photosystem II protein, ATP synthase, and ribulose bisphosphate carboxylase. Lauritano et al. [43] analyzed the changes in transcripts when *T. suecica* was cultivated in the presence and absence of nitrogen, observing numerous changes in their regulation such as an up-regulation in transcripts related to stress response or solute transport, and a down-regulation in transcripts related to amino acid synthesis or photosynthetic activity. Although they did not evaluate protein expression directly, these changes in transcript expression were indirectly related to changes in protein expression (even though the latter does not depend exclusively on transcripts). Consequently, this research confirmed that culture conditions are a key determinant of the molecular and protein expression in microalgae.

### 2.3. Optimization of the Soluble Protein Extraction Process

In order to extract the soluble proteins present in both consortia, the solubilization of the biomasses was carried out at two different pH levels (10 and 12) and the subsequent protein precipitation was evaluated using three different pH levels (3, 4, and 5). Supernatants and pellets were separated by centrifugation, and the soluble protein content was measured in the supernatants (Table 2). Protein results are expressed as % (g of protein in 100 g of dw of supernatant).

Although in both biomasses the solubilization pH affected the protein content of the soluble fraction, being higher when proteins were solubilized at pH 10, the differences were more remarkable for C1. This could be due to the higher protein content of the initial biomass. At both solubilization pHs, the protein content of the supernatants obtained from C1 was higher when precipitation was carried out at pH 5, reaching 30.59%. This value indicated that, with these conditions, the protein recovery achieved was 62.88% (Table 1 and Table 2). Based on these results, a pH 10–pH 5 combination was chosen as the optimal protein solubilization and precipitation pHs for both biomasses. This choice was made according to the protein content of supernatants and the efficiency of the process in terms of time and material used. With these optimal conditions selected, the protein extraction process was repeated 18 times to generate enough material for subsequent tests, obtaining similar results, with a protein content of 28.50 ± 2.69% (C1) and 8.46 ± 0.45% (C2). The extraction yields achieved, expressed as % (g of dw of extract in 100 g of dw of biomass), were 25.89 ± 3.67 and 25.07 ± 1.30% for C1 and C2, respectively.

### 2.4. Hydrolysis of the Soluble Protein Extract with Alcalase

**Figure 1:**
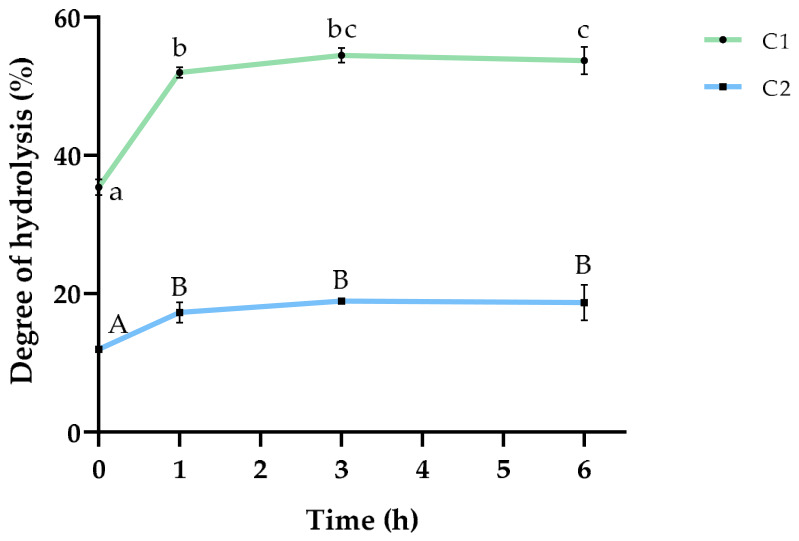
Evolution of the degree of hydrolysis during the incubation of soluble proteins extracted from consortia biomass C1 (green) and C2 (blue) with Alcalase 2.4 L. Results are expressed as mean ± standard deviation. ^a–c; A–B^: Different letters indicate significant differences over time (*p* < 0.05); lowercase letters for C1 and uppercase letters for C2.

The supernatants were hydrolyzed with Alcalase 2.4 L at the optimal enzyme conditions (pH = 8.5 and 50 °C) and an enzyme:substrate (E:S) ratio of 10% (*w*/*w*). The DH was measured at the beginning of the hydrolysis and after 1, 3, and 6 h of incubation with the enzyme. The results are shown in Figure 1.

In both biomasses, hydrolysis was observed at the start point of the reaction (t = 0 h), with DH values of 34.86 ± 1.16% for C1 and 12.27 ± 0.38% for C2 (Figure 1). This indicated that during the extraction process, hydrolysis of proteins could occur [44]. After incubation with the proteolytic enzyme, the DH increased, reaching 54.55 ± 2.18% and 19.51 ± 2.18% after 6 h for C1 and C2, respectively. The low DH for the hydrolysate obtained from the C2 biomass could be due to the resistance of the proteins contained in this consortium to the action of the enzyme. Moreover, for this biomass, hydrolysis only occurred during the first hour of incubation, indicating that additional incubation with Alcalase 2.4 L was not effective to further hydrolyze proteins contained in C2. However, although the highest DH increase was observed during the first hour of incubation of C1 proteins with Alcalase 2.4 L, this enzyme was able to further hydrolyze, reaching the highest DH after 3 h of incubation.

Shishavan et al. [45] reported a DH of 25.08 ± 0.75% when the proteins present in *Arthrospira* spp. were incubated with Alcalase. Also, the highest increase in the DH was observed during the first hour of reaction, which is consistent with the results obtained in our study for C1. Akbarbaglu et al. [44] studied the hydrolysis with Alcalase 2.4 L of *A. platensis* proteins in combination with ultrasonic pre-treatment, reaching a DH of 29.2% after 1 h of reaction. Zhang et al. [14] observed that, among several enzymes used, Alcalase hydrolyzed proteins up to 21% after 5 h of incubation. Recently, Pekkoh et al. [46] reported values around 25% of DH of *Chlorella* spp. proteins after 20 and 30 min of hydrolysis with Alcalase 2.4 L at 50 °C, assisted by ultrasounds. In comparison, our results show that the DH for C1 was higher, when compared to previous research conducted with individual microorganisms contained in this consortium.

Regarding the species present in C2, there have been no previous studies carried out with *Kamptonema* spp. proteins. Md Saleh et al. [47] carried out an optimization study using response surface methodology, in which a maximum DH of 55.76% was achieved when proteins from *N. gaditana* were hydrolyzed by Alcalase 2.4 L. Thus, further research is encouraged to evaluate the effect of Alcalase and other food grade enzymes on the DH of less common but potentially bioactive microalgae species.

### 2.5. Characterization of Protein Hydrolysates with Alcalase

The contents of protein, total phenolic compounds, and chlorophylls a and b were determined in the supernatants obtained after the hydrolysis of protein extracts with Alcalase 2.4 L at different incubation times (Table 3). Protein results are expressed as % (g of protein in 100 g of dw of supernatants), phenolic compounds are expressed as mg GAE per g of dw of supernatant, and chlorophylls are expressed as mg of chlorophyll per g of dw of supernatant.

A decrease in the protein content of C1 supernatant was observed within the first 3 h of Alcalase 2.4 L incubation, with levels remaining unchanged between 3 and 6 h. In contrast, the protein content of the C2 supernatant remained constant throughout the hydrolysis. Within 1 h, enzymatic incubation significantly boosted total phenolic compounds in the hydrolysates, with no further increase observed thereafter. The final concentrations were approximately 16 mg GAE/g sample and 6 mg GAE/g sample for C1 and C2, respectively, with no significant differences observed between 1, 3, and 6 h of hydrolysis. The increase in these compounds during hydrolysis could be related to their release from complexes with proteins after the action of the enzyme. These complexes result from the interaction between hydrophobic amino acids (such as leucine, alanine, or cysteine) and the nonpolar aromatic rings of phenolic compounds [48].

C1 showed an initial amount of chlorophyll a and b of 1.15 mg/g sample and 2.31 mg/g sample, respectively. In the case of C2, the values were 0.85 mg/g sample and 1.52 mg/g sample for chlorophyll a and b, respectively. In C1, the content of both pigments decreased during the first 3 h of hydrolysis, with no changes at longer times of incubation with the enzyme. In the case of C2, the levels of both chlorophylls were reduced during Alcalase 2.4 L hydrolysis. Within chloroplasts, chlorophylls are stabilized by their integration into protein complexes as part of the photosynthetic system [49]. Free chlorophyll, however, is highly sensitive to degradation under high temperature, low pH, and enzymatic reactions [50]. Therefore, the hydrolysis of photosynthetic proteins during our process would have released chlorophylls from their protective complexes, exposing them to degradative conditions and leading to a decrease in pigment levels.

**Figure 2:**
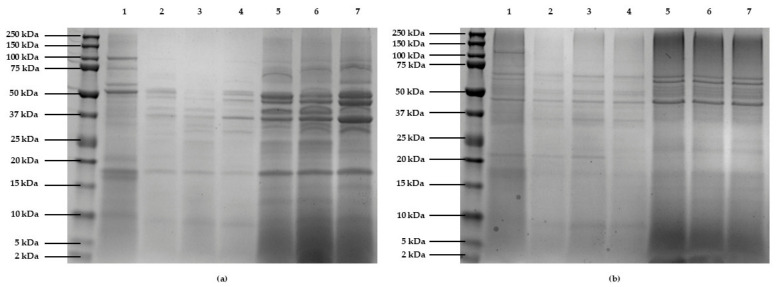
SDS-PAGE gels of the hydrolysates obtained from (**a**) C1 biomass and (**b**) C2 biomass. (1) Protein extract obtained from biomasses at a solubilization pH of 10 and precipitation pH of 5. (2, 3, 4) Supernatants obtained from the Alcalase 2.4 L hydrolysis of biomass proteins for 1, 3, and 6 h, respectively. (5, 6, 7) Pellets from the Alcalase 2.4 L hydrolysis of biomass proteins for 1, 3, and 6 h, respectively.

The protein profiles after hydrolysis were analyzed by polyacrylamide gel electrophoresis with sodium dodecyl sulfate (SDS-PAGE, Figure 2). In the C1-derived protein extract, bands between 10 and 250 kDa were observed (Figure 2a). The intensity of these bands was reduced in the supernatants after Alcalase 2.4 L hydrolysis due to proteolytic degradation. However, the pellets contained persistent high molecular weight proteins resistant to enzymatic action, as well as a band corresponding to Alcalase 2.4 L itself (~27 kDa). The hydrolysis time course showed similar protein profiles in both fractions at all time points, indicating that brief incubation with this microbial enzyme was adequate to degrade the susceptible microalgae proteins, with no further effect from extended incubation. In the protein extract obtained from C2, bands between 10 and 120 kDa were observed (Figure 2b). The results obtained were similar to those obtained in C1, with a reduction in the intensity or disappearance of high molecular weight proteins in the hydrolysate samples. Among the different hydrolysis times, no clear differences were observed in the protein profiles, with a decrease in the intensity of some bands in the hydrolysate after 6 h of incubation. In general, the C2 gel showed more diffuse and less dense bands compared to C1, which could be attributed to the lower protein content of this biomass. Finally, in both C1 and C2 hydrolysates, bands of <10 kDa were observed, which could correspond to small peptides released during hydrolysis.

### 2.6. Antioxidant Capacity of the Alcalase Hydrolysates

**Figure 3:**
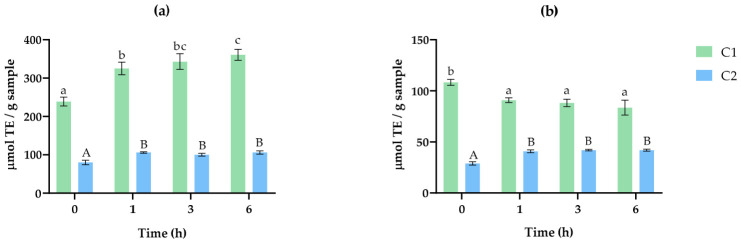
Antioxidant capacity measured using (**a**) ORAC and (**b**) ABTS of the hydrolysates obtained after hydrolysis with Alcalase 2.4 L at 0, 1, 3, and 6 h of the protein extract of C1 (green) and C2 (blue) obtained under optimal protein extraction conditions (pH 10–pH 5). TE: Trolox equivalents. Results are expressed as mean ± standard deviation. ^a–c; A–B^: Different letters indicate significant differences (*p* < 0.05); lowercase letters for C1 and uppercase letters for C2.

The antioxidant capacity of Alcalase 2.4 L hydrolysates was measured using the Oxygen Radical Absorbance Capacity (ORAC) and 2.2′-Azino-bis (3-ethylbenzothiazoline-6-sulfonic acid) diammonium salt (ABTS) radical assays. The results are shown in Figure 3a, b. The results demonstrated that both microalgae-cyanobacteria consortia presented antioxidant capacity through two different mechanisms of action, neutralizing both peroxyl and ABTS radicals. However, lower values were obtained in the hydrolysates from C2 when compared to C1. The antioxidant capacity could be related to peptides released by Alcalase 2.4 L action on microalgae and/or cyanobacteria proteins, as demonstrated by the increase in DH. However, other compounds, such as phenolic compounds present in the hydrolysates and whose content increased during hydrolysis, could also have contributed to the effects observed. In this regard, Agregan et al. reported values of 0.20 ± 0.00 g phloroglucinol equivalents (PGEs)/100 g dw for *A. platensis* and 0.35 ± 0.00 g PGE/100 g dw for *C. vulgaris*, while Alzahrani et al. reported values of 0.60 ± 0.05 GAE mg/100 g for *A. platensis* and 0.28 ± 0.01 GAE mg/100 g for *C. vulgaris* [51,52].

The initial biomass of C1 already showed antioxidant capacity, with an ORAC value of 235.40 ± 12.44 μmol Trolox equivalents (TE)/g sample. This value increased significantly after Alcalase 2.4 L hydrolysis, reaching 360.88 ± 16.64 μmol TE/g after 6 h of reaction (Figure 3a). In the case of the ABTS assay (Figure 3b), the highest antioxidant value was obtained at 0 h (108.28 ± 3.30 μmol TE/g sample) for C1, which decreased after the action of Alcalase 2.4 L after 1 h of incubation (90.83 ± 2.69 μmol TE/g sample). This reduction in ABTS radical-neutralizing activity could be related to the degradation of potential BAPs present in the initial biomass, thus losing their ability to donate electrons or hydrogen [45,52].

In the C2-derived hydrolysates, ORAC and ABTS activities were lower than that observed for C1 hydrolysates (Figure 3a,b). This might be attributed to differences in protein structure, quantity, and amino acid composition, which could yield less potent antioxidant peptides upon hydrolysis. In addition, the higher content of phenolic compounds in C1 hydrolysates could also contribute to the higher antioxidant capacity observed. A significant increase in ORAC activity was observed after the action of Alcalase 2.4 L, with a value of 80.07 ± 6.83 μmol TE/g sample at time 0 h and 106.12 ± 2.01 μmol TE/g sample after the first hour of hydrolysis, with no significant differences at longer incubation times. For the ABTS assay, the results showed the same trend, with values increasing from 28.94 ± 1.92 μmol TE/g sample (0 h) to 40.81 ± 1.62 μmol TE/g sample (1 h), with no further significant changes at longer times.

The antioxidant capacity increase after the incubation of consortia biomass proteins with Alcalase 2.4 L indicates that peptides released from the action of this proteolytic enzyme could be the major mechanism responsible, as has been previously reported [45]. The study of Otero and Verdasco-Martín [53] also reported high ORAC values for *A. platensis* hydrolysates with Alcalase. The higher ORAC values reported by Cunha et al. [24] for the cellulase-protease hydrolysis of *C. vulgaris*, compared to our findings, suggest that cellulase pre-treatment could facilitate the liberation of intracellular proteins, thereby increasing the substrate available for protease to generate antioxidant peptides.

### 2.7. Exploratory Data Analysis and Clustering

**Figure 4:**
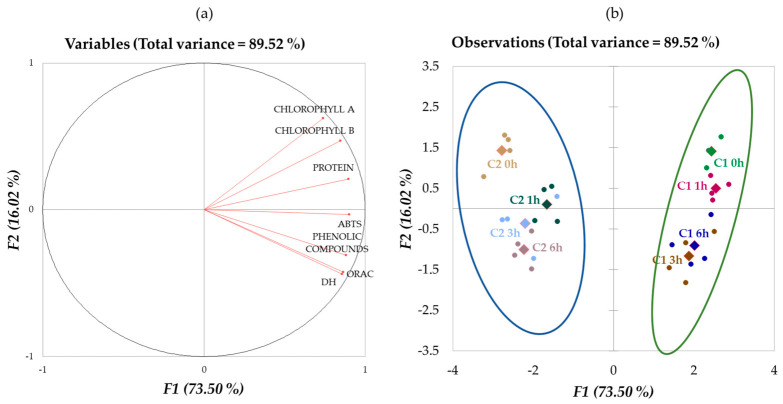
PCA ((**a**), loading plot; (**b**), score plot) of the results of hydrolysis with Alcalase 2.4 L of consortia C1 (green ellipse) and C2 (blue ellipse) at different times (0, 1, 3, and 6 h). The centroids (different colored diamonds) group the quadruplicates of the hydrolyzed samples at the same hydrolysis time.

To obtain an overview of the main factors that affect the antioxidant capacity of the consortia biomass hydrolysates, a principal component analysis (PCA) was carried out with all the data (Figure 4a,b). The first two principal components accounted for 89.52% of the total variance.

The two consortia biomasses (C1 and C2) were clearly discriminated along F1, while F2 segregated the samples according to hydrolysis time. The C1 hydrolysates showed positive values of F1, while the C2 hydrolysates showed a negative response. This is consistent with the higher protein, phenolic compound and chlorophyll contents, with DH and antioxidant capacity described above for C1. Regarding F2, the centroids results confirm the differences between the samples depending on the hydrolysis time, with higher levels of chlorophyll and protein at time 0 and 1 h, and lower DH, phenolic compounds, and ORAC activity values when compared to 3 and 6 h in both biomasses.

Figure 4a confirmed that ORAC values increased as hydrolysis time and phenolic compound content increased. In contrast, the ABTS measurement did not seem to have a clear influence; thus, the values obtained would not be so closely related to DH or to the presence of phenolic compounds in the hydrolysates. These results indicated that the increase in antioxidant capacity measured by the ORAC method was mainly related to the presence of phenolic compounds and the DH, while protein and chlorophyll content had less influence. The increase in antioxidant capacity occurred mainly during the first hour, without a notable increase at longer times. Similarly, phenolic compounds increased during the first hour of hydrolysis. Our results were consistent with previous studies that support this relationship between antioxidant capacity and the presence of phenolic compounds [51,52]. In addition, the work of Alzahrani et al. [52] and Otero and Verdasco-Martín [53] showed that hydrolysates obtained with Alcalase had the highest correlation between the presence of phenolic compounds and antioxidant capacity, possibly demonstrating the ability of this enzyme to release phenolic compounds from existing complexes.

**Figure 5:**
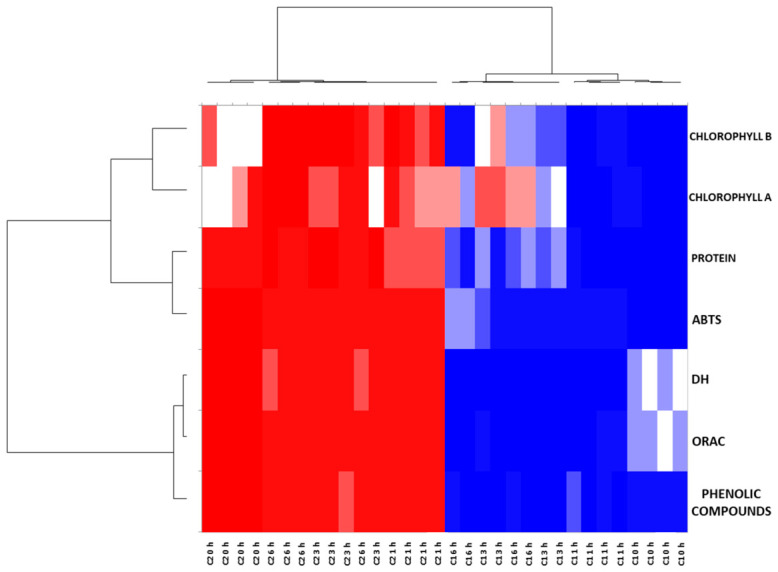
Heat map and agglomerative hierarchical cluster (AHC) of the results of consortia C1 and C2 protein hydrolysis with Alcalase 2.4 L at different times (0, 1, 3, and 6 h). The correlation between samples and variables is represented on a scale from red (lowest correlation, −1) to blue (highest correlation, +1).

The heat map and the agglomerative hierarchical cluster (AHC) dendrogram obtained are presented in Figure 5. The correlation between variables and samples is represented on a scale from red (least correlated, −1) to blue (most correlated, +1). The upper dendrogram grouped the samples according to the two consortia and perfectly differentiated between C1 and C2. Finally, in the lateral dendrogram, it can be observed that protein content is correlated with ABTS and that the variables DH, ORAC, and phenolic compounds are grouped, confirming the relationship between them.

### 2.8. In Silico Hydrolysis of Identified Proteins and Prediction of Antioxidant Activity

To estimate the release of potential antioxidant peptides by Alcalase 2.4 L, an in silico hydrolysis was performed on 1428 and 866 proteins identified in the C1 and C2 consortia, respectively. After removing duplicate peptides and free amino acids, a total of 15,799 and 21,325 unique peptides were identified in C1 and C2, respectively. Among these, 7142 peptide sequences were common to both biomasses. The proteins that released more than 50 peptides were selected for further studies. Thus, 1001 proteins from C1 released 3783 peptides (3194 from *A. platensis* and 589 from *C. vulgaris*) and 85 proteins from C2 released 5773 peptides (3766 from *Kamptonema* sp., 1804 from *C. vulgaris*, and 203 from *N. oculata*). Of these proteins, those that released the highest number of peptides during hydrolysis of C1 were alanyl-tRNA synthetase from *A. platensis* (132 peptides) and acetyl-CoA carboxylase from *C. vulgaris* (130 peptides). Regarding C2, proteins that produced the highest number of peptides were RNA helicase from *C. vulgaris* (151 peptides), followed by putative D-alanine-poly (Phosphoribitol) ligase from *Kamptonema* sp. (139 peptides) and the elongation factor G, mitochondrial from *C. vulgaris* (122 peptides).

**Figure 6:**
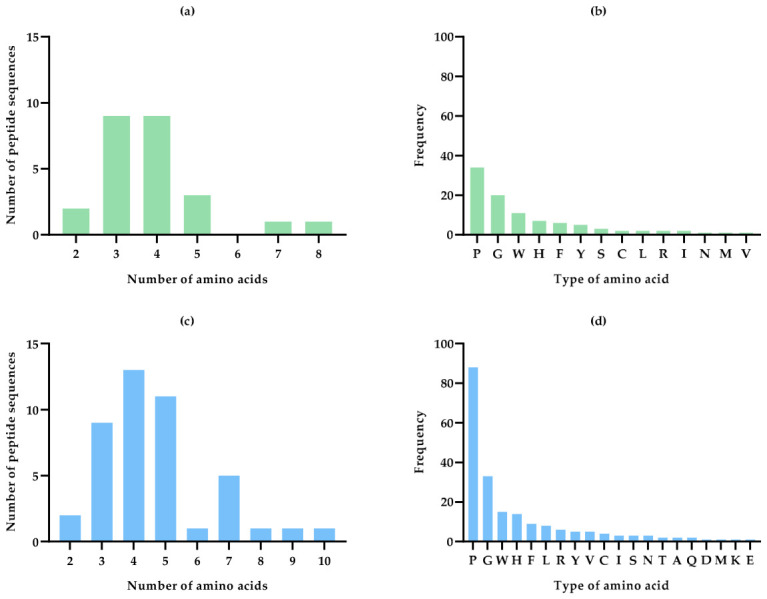
Peptide sequence analysis using AnOxPP and AnOxPePred databases that would demonstrate potential antioxidant activity from C1 (**a**,**b**) and C2 (**c**,**d**). (**a**,**c**) Peptide classification by the number of amino acids; (**b**,**d**) peptide classification by the type of amino acids and the frequency with which they are present.

Selected peptides (3783 for C1 and 5773 for C2) were ranked using the Peptide Ranker software, considering the potential bioactivity of those peptides with a score of ≥0.8. Thus, 221 peptides for C1 (184 from *A. platensis* and 37 from *C. vulgaris*) and 328 peptides for C2 (199 from *Kamptonema* sp., 124 from *C. vulgaris*, and five from *N. oculata*) were selected to estimate their potential antioxidant activity by using the AnOXPP and AnOxPePred databases. A total of 177 peptides from C1 and 243 from C2 were estimated to have antioxidant activity with the AnOxPP database. Regarding the AnOxPePred database, 28 peptide sequences from C1 and 51 peptide sequences from C2 seemed to have scavenging activity. By comparing the sequences that reported antioxidant activity in both databases, a total of 25 and 44 common peptide sequences with antioxidant activity were obtained for C1 and C2, respectively. The potential toxicity of these peptides was evaluated using the software ToxinPred, which was also useful in providing a general characterization of the physicochemical characteristics of these sequences (Supplementary Table S3). In addition, a deeper analysis of the sequences was carried out to get an overview of the number, type, and frequency of the amino acids of C1 and C2. As shown in Figure 6a and Figure 6c, peptides containing between three and five amino acids represent 84 and 75% of the total potential antioxidant peptides identified in C1 and C2 hydrolysates, respectively.

Additionally, the proportion of hydrophobic amino acids in the identified sequences was 84.5% for C1 and 79.6% for C2. The predominant amino acids were phenylalanine (F), proline (P), glycine (G), and tryptophan (W). These results are related to the type of enzyme used, since Alcalase is an enzyme typically intended for the production of hydrophobic peptides due to its preferred cleavage sites [54]. Several studies have demonstrated the relationship between the presence of hydrophobic amino acids (F, P, W, valine (V) or leucine (L)), aromatic amino acids (W or tyrosine (Y)), and sulfur-containing amino acids (cysteine (C) or methionine (M)), their position within the peptide chain, such as whether the amino acids are located at the N-terminal or C-terminal positions of the peptide sequence, and the antioxidant activity [55]. These structural characteristics give them the ability to donate electrons or hydrogen atoms, thus neutralizing free radicals, as well as the ability to chelate metals or increase their solubility in the medium, thereby increasing their antioxidant effect [55,56,57]. This effect is due to their action against free radicals, acting like electron donors and interacting with them [56,57].

The peptide sequences predicted to be antioxidant by both databases (25 from C1 and 44 from C2) were evaluated by using the BIOPEP-UWM database. Sequences described as bioactive are shown in Table 4.

Seven sequences had previously been reported in the literature for their bioactive properties. Of them, peptides GW and PW (identified in both consortia hydrolysates), and PHF and PHW (identified in C1) have been described as antioxidant peptides. The synthetic peptide GW was reported as a potent radical-neutralizing agent measured by ORAC and DPPH assays [58]. The antioxidant activity was attributed to the presence of W within the sequence. Moreover, antihypertensive, antidiabetic, and peptidase inhibitory properties of this dipeptide were also reported in the literature [59,60,61]. PW is a potent antioxidant and antidiabetic dipeptide released from buckwheat protein isolate after its simulated gastrointestinal digestion with pepsin and pancreatin [60,62]. Similarly, synthetic peptides PHF and PHW were reported by Saito et al. [67] for their antioxidant properties mediated through radical scavenging activity. In addition, tripeptide PPW is included within the sequence of peptides FPPWVL and FPPWF identified in *A. platensis* hydrolysates and reported as antioxidant [66].

The released peptides during Alcalase 2.4L hydrolysis could be related to the increased antioxidant capacity, as shown in Figure 3. Although the in silico analysis predicted a higher number of potential antioxidant peptides released from C2 proteins in comparison with those released from C1, the lower in vitro activity obtained for the C2 hydrolysates could be associated with the concentration of peptides or the possible synergies or antagonisms resulting from the interaction between peptides or between peptides and other compounds. The divergence between the in vitro and in silico results could likely be attributable to a combination of factors, highlighting the indispensable role of experimental validation in verifying in silico predictions of potential bioactive peptides from microalgae-cyanobacteria consortia. Overall, the bioactive capacity of the hydrolysates derived from both biomasses shows the industrial potential of these microorganisms, providing a novel and sustainable alternative for bioactive compounds with antioxidant activity.

## 3. Materials and Methods

### 3.1. Samples and Reagents

Two microalgae-cyanobacteria consortia biomasses, C1 and C2, were supplied by Microalgae Solutions S.L. (Madrid, Spain). These marine consortia were cultivated under controlled conditions in flat photobioreactors at 25 °C, with a photoperiod of 16:8 and a photon flux density of 100 µmol/m^2^s, in modified Guillard F/2 medium [4] following a patented process (ES2673369) [72], freeze-dried, and stored in the dark at 4 °C until analysis. C1 was a consortium constituted of *C. vulgaris* and *A. platensis*. C2 was a consortium comprising *Kamptonema* sp., *N. oculata*, *T. suecica,* and *C. vulgaris*. Sodium hydroxide (NaOH), citric acid, hydrochloric acid (HCl), o-phthaldialdehyde (OPA), L-serine, 6-hydroxy-2.5.7.8-tetramethylchromane-2-carboxylic acid (Trolox), ABTS, Folin–Ciocalteu’s reagent and gallic acid were obtained from Sigma-Aldrich (Burlington, MA, USA). Alcalase^®^ 2.4 L FG (2.4 AU-A/g), which is a serine endopeptidase derived from *Bacillus licheniformis*, was purchased from Strem Chemicals, Inc. (Newburyport, MA, USA).

### 3.2. Characterization of Microalgae-Cyanobacteria Consortia

The protein content of C1 and C2 biomasses was determined by Kjeldahl (981.10 method, AOAC International) [73] using a block digester (J.P. Selecta, Barcelona, Spain) and a Buchi Kjeldahl K-314 distillation unit (BÜCHI Labortechnik AG., Flawil, Switzerland). A conversion factor of 5.95 was used to convert the nitrogen into protein [74]. Protein content was also determined by the BCA method, using the Thermo Scientific^TM^ Pierce^TM^ BCA commercial kit (Waltham, MA, USA) and bovine serum albumin (BSA) as standard (25–1000 μg/mL). Absorbance was measured at 562 nm on a BioTek Synergy^TM^ HT plate spectrophotometer (Winooski, VT, USA). Results were expressed as μg protein/mL and protein percentage. Samples were analyzed in triplicate.

The total lipid content was determined by conventional Folch extraction, following Figueiredo et al. [75], and expressed as extraction yield (% *w*/*w*). This determination was carried out in triplicate. Additionally, total carbohydrate content was determined by sulfuric acid-ultraviolet spectrophotometric assay developed by Albalasmeh et al. [76], using glucose as a standard (0–0.20 mg/mL). Results were expressed as a percentage of total carbohydrate in the sample. Samples were analyzed in triplicate. Moisture analysis of the samples was performed by drying the samples to constant weight. The results were expressed as a percentage of the weight of the dry extract obtained. The samples were analyzed in duplicate. Inorganic material analysis was carried out based on the AOAC International Ash 923.03 method [77]. Samples were analyzed in duplicate, and results were expressed as a percentage of ash in the sample.

### 3.3. Identification of Proteins Present in the Microalgae-Cyanobacteria Consortia

Consortia biomasses were first suspended in 50 µL of sample buffer before being applied to 1.2 cm-wide wells of a traditional SDS-PAGE gel (0.75 mm thick, 4% stacking and 10% resolving; Bio-Rad, Hercules, CA, USA). When the front got 3 mm into the resolving gel, the run was stopped. The whole proteome was then concentrated in the stacking/resolving gel interface. Coomassie staining (Bio-Rad) was used to observe the unseparated protein bands, which were removed, cut into 2 × 2 mm cubes and put in 0.5 mL microcentrifuge tubes [78]. The gel fragments were destained using acetonitrile:water (ACN:H_2_O, 1:1), subjected to reduction and alkylation (disulfide bonds from cysteine residues were reduced with 10 mM dithiothreitol (DTT) for 1 h at 56 °C, followed by alkylation of thiol groups with 10 mM iodoacetamide for 30 min at room temperature in darkness), and subsequently digested in situ with sequencing grade trypsin (Promega, Madison, WI, USA) as outlined by Shevchenko et al. [79], with minor modifications. By employing enough ACN to remove all liquid, the gel pieces were shrunk. The gel fragments were dried in a speedvac after the ACN was pipetted out. The dried gel fragments were re-swollen in 100 mM Tris-HCl pH 8, 10 mM calcium chloride (CaCl_2_) and 60 ng/µL trypsin at a protein:enzyme (*w*/*w*) ratio of 5:1. The tubes were incubated at 37 °C for 12 h after being placed on ice for 2 h. A total of 0.2% RapiGest (Waters, Milford, MA, USA) was present during the digestion process. Finally, 1% trifluoroacetic acid (TFA) was added to stop digestion. Prior to the mass spectrometric measurement, whole supernatants were dried and desalted onto OMIX Pipette tips C18 (Agilent Technologies, Santa Clara, CA, USA).

The desalted protein digests were then dried, resuspended in 10 µL of 0.1% formic acid and subjected to RP-LC-MS/MS analysis in an Easy-nLC 1200 system coupled to an ion trap LTQ-Orbitrap-Velos-Pro hybrid mass spectrometer (Thermo Scientific, Waltham, MA, USA). The peptides were concentrated (online) by reverse phase chromatography using a 0.1 mm × 20 mm C18 RP precolumn (Thermo Scientific) and then separated using a 0.075 mm × 250 mm bioZen 2.6 µm Peptide XB-C18 RP column (Phenomenex, Torrance, CA, USA) at 0.25 μL/min. A 180 min dual gradient was used to elute the peptides. The gradient profile (solvent A: 0.1% formic acid in water, solvent B: 0.1% formic acid, 80% ACN in water) was set at 5−25% solvent B for 135 min, 25−40% solvent B for 45 min, 40−100% solvent B for 2 min, and 100% solvent B for 18 min. A Nano-bore emitter Stainless Steel ID 30 μm (Proxeon, Odense, Denmark) interface was used for ESI ionization at a spray voltage of 2.1 kV with a 60% S-Lens. The Orbitrap resolution was 30.000 [80]. Following twenty data-dependent MS/MS scans (Top 20) with an isolation width of 2 u (in mass-to-charge ratio units), a normalized collision energy of 35% and dynamic exclusion performed for 60 s intervals, peptides were detected in survey scans ranging from 400 to 1600 amu (1 μscan). Rejecting unassigned and singly charged protonated ions was made possible by charge-state screening.

Finally, peptide identification from raw data was carried out using the PEAKS Studio v11.5 search engine (Bioinformatics Solutions Inc., Waterloo, Ontario, Canada). A database search was performed against UniProt-*A. platensis* + UniProt-*C. vulgaris* for C1 and UniProt-*Kamptonema* sp. + UniProt-*N. oculata* + UniProt-*T. suecica* + UniProt-*C. vulgaris* for C2 (decoy-fusion database). The searches were conducted with the following limitations: tolerances of 20 ppm for precursor ions and 0.6 Da for MS/MS fragment ions, tryptic cleavage after arginine and lysine (semi-specific), and up to two missed cleavage sites. The searches were performed allowing optional methionine oxidation and cysteine carbamidomethylation. False discovery rates (FDRs) for PSM and for protein was set to 0.01. Proteins were deemed reliably identified only if at least two distinct peptides were found using LC/MS/MS analysis [78,81,82,83].

The mass spectrometry proteomics data have been deposited in the ProteomeXchange Consortium via the PRIDE [84] partner repository with the dataset identifier PXD077201 and 10.6019/PXD077201 for C1 and PXD077149 and 10.6019/PXD077149 for C2.

### 3.4. Optimization of the Extraction Process of Soluble Protein from Consortia Biomasses

Optimization of the protein extraction of C1 and C2 biomasses was carried out by testing two solubilization pH levels (10 and 12) and three precipitation pH levels (3, 4, and 5). For this purpose, 500 mg of lyophilized biomasses were dissolved in 5 mL of distilled water, and the pH was adjusted to 10 or 12 with 6 M NaOH. The solution was then incubated in a BÜCHI Heating Bath B-490 at 50 °C for 2 h. After incubation, the pH of the solution was decreased with 3 M citric acid to pH 3, 4 or 5 to achieve protein precipitation. Then, samples were centrifuged in Eppendorf^TM^ Centrifuge 5804 R (Hamburg, Germany) for 10 min at 4 °C and 4500 g to separate the supernatant from the pellet, which were lyophilized and stored at −20 °C for further analysis. All extractions were carried out in triplicate. The protein content of the supernatants was determined by the BCA assay, as previously described (Section 3.2).

### 3.5. Enzymatic Hydrolysis of the Soluble Protein Extract

The enzymatic hydrolysis of the soluble proteins obtained following the final selected extraction conditions was carried out using Alcalase 2.4 L as the proteolytic enzyme. Samples (1 g) were dissolved in 50 mL of distilled water, setting the pH to 8.5 with NaOH 6 M and the temperature at 50 °C. The enzyme was added to the sample using an E:S ratio of 10% (*w*/*w*), and the reaction took place for 6 h at 50 °C in a BÜCHI Heating Bath B-490. Aliquots were taken immediately after adding the enzyme (0 h), and after 1, 3, and 6 h of incubation. Hydrolysis conditions were selected in agreement with optimal conditions of the enzyme used and based on the existing literature [45]. The enzyme was inactivated by heating at 100 °C for 10 min. Part of the aliquots were separated to determine the DH, and the other part was centrifuged in a Hettich^TM^ Zentrifugen Universal 320R centrifuge (Tuttlingen, Germany) for 20 min at 20 °C and 10,000 rpm, separating the supernatant from the pellet, which were lyophilized and preserved at −20 °C for further analyses. The hydrolysis was performed in duplicate.

### 3.6. Characterization of Alcalase Hydrolysates

#### 3.6.1. Determination of the Degree of Hydrolysis

The DH of the hydrolysates was determined using the OPA method, according to the protocol described by Nielsen et al. [85], and compared with the result from the total acid hydrolysis of the proteins, carried out with HCl 6 M. For the standard curve, L-serine was prepared in Milli-Q water and used at concentrations between 0 and 0.2 mg/mL. Absorbance was measured at 340 nm in a BioTek Synergy^TM^ HT plate spectrophotometer. The absorbance result obtained for the samples was interpolated into the standard curve, and the results were expressed in mg/mL of L-serine. The analysis was performed in duplicate. DH was calculated as follows:

DH(%)=(h/HT)×100,

where h is the concentration of free amino groups in each sample, and HT is the total number of free amino groups per protein equivalent.

#### 3.6.2. Protein Profile by SDS-PAGE Electrophoresis Gel

Protein and peptide profiles of the pellets and supernatants resulting from the hydrolysis of C1 and C2 soluble protein extracts were analyzed by SDS-PAGE. Samples were prepared in the sample buffer and incubated at 100 °C with agitation for 5 min in an Eppendorf^TM^ Thermomixer (Hamburg, Germany). The electrophoresis was performed on the automated equipment Criterion (Bio-Rad) using polyacrylamide 12% Bis-Tris Criterion^TM^ XT Precast Gels (Bio-Rad). A total of 35 μL of each sample (containing 50 μg of protein) and 10 μL of the standard Precision Plus Protein™ Dual Xtra Prestained Protein Standards (Bio-Rad) were loaded. Commercial buffer XT MES Running Buffer 20X (Bio-Rad) was used for separation. After the electrophoretic migration, gels were washed with Milli-Q water and fixed with 40% methanol and 20% acetic acid solution for 30 min in the dark. Subsequently, gels were stained with BlueSafe Coomassie NZYTech (Lisbon, Portugal) and washed again with Milli-Q water. Finally, fresh water was added, and the gels were left in agitation at 4 °C overnight. Images of the gels were taken using the gel reader Versadoc Imaging System and processed with Image Lab 6.1 software (Bio-Rad).

#### 3.6.3. Determination of Total Chlorophylls and Carotenoids

Analysis of chlorophyll a and b and total carotenoids of the hydrolysates was performed according to the protocol described by Lichtenthaler and Buschmann [86] and Maadane et al. [87]. Absorbance was measured at wavelengths of 470, 652, and 665 nm in the plate reader BioTek Synergy^TM^ HT. The results of chlorophyll a, b, and carotenoids were expressed in mg/g extract. Samples were analyzed in duplicate. Contents were calculated as follows:

Chlorophyll a = 16.72 × A665 − 9.16 × A652;

Chlorophyll b=34.09×A652−9.16×A665;

Carotenoids=(1000×A470−1.91×Chlorophyll a−95.15×Clorophyll b) / 225.

#### 3.6.4. Determination of Total Phenolic Compounds

Analysis of total phenolic compounds of the hydrolysates was performed through the Folin–Ciocalteu method, using the protocol described by Singleton et al. [88]. Gallic acid was used as a standard, at concentrations between 0.05 and 1.0 mg/mL. Absorbance was measured at 760 nm in the plate reader BioTek Synergy^TM^ HT. Results were expressed as μg GAE/mg fraction. The analysis was performed in duplicate.

### 3.7. Determination of the Antioxidant Capacity of the Hydrolysates

Antioxidant capacity of the hydrolysates was measured using the ORAC assay, following the method described by Hernández-Ledesma et al. [89]. Trolox solution was used as a standard, at concentrations between 1 and 8 μM. Fluorescence was measured every minute at an excitation wavelength of 485 nm and an emission wavelength of 520 nm. With the values obtained, the area under the curve (AUC) was calculated and expressed as a function of the amount of Trolox (μmol) or sample (g). The ORAC value, expressed in μmol TE/g of sample, was obtained by dividing the value of the sample slope by the value of the Trolox slope. Samples were analyzed in duplicate.

Antioxidant capacity of the hydrolysates was also measured using the ABTS radical neutralization assay [90]. Trolox solution was used as a standard, at concentrations between 25 and 200 μM. Absorbance at 734 nm was measured in the plate reader BioTek Synergy^TM^ HT. The absorbance values obtained for the samples were interpolated to the Trolox standard curve, and the results were expressed as μmol TE/g sample. Samples were analyzed in duplicate.

### 3.8. In Silico Hydrolysis and Antioxidant Activity Prediction

In silico hydrolysis of the proteins present in both consortia biomasses, previously identified as detailed in Section 3.3, was carried out using the Rapid Peptides Generator (RPG) software v22.2.3 [91], which predicts the cleavage sites of the enzymes in the protein sequences. Peptide sequences were generated using Alcalase, which was previously designed and installed in the software following the grammar indications of the user’s guide. After in silico hydrolysis, the peptide sequences generated were evaluated in the Peptide Ranker tool by Bioware [92], which predicts the probability of a peptide to be bioactive, following the criteria selection of peptides with a score ≥ 0.8. Finally, the antioxidant activity estimation of peptides with a higher value or equal to 0.8 after Peptide Ranker analysis was performed using the AnOxPP-1.0 and AnOxPePred-1.0 software [93,94]. Toxicity and general physicochemical characteristics of the selected sequences were evaluated with ToxinPred 1.0 software [95], and their bioactivity was screened and compared with the bioactive peptide sequences reported using BIOPEP-UWM [96].

### 3.9. Statistical Analysis

Statistical analysis of the results was carried out by means of a one-factor analysis of variance (ANOVA) using the IBM SPSS Statistics 28.0 program (Chicago, IL, USA). Statistical significance was established for *p* < 0.05 for all cases. Exploratory analysis of the results was performed by PCA, AHC and heat map, using the XLSTAT Premium 2018.5.53172 software (Addinsoft, Paris, France).

## 4. Conclusions

This study demonstrates that the biomasses evaluated, composed of different microalgae and cyanobacteria species, are sustainable sources of high-value proteins. The protein recovery process was optimized by solubilization at pH 10 and precipitation at pH 5, followed by optimized protein hydrolysis with Alcalase 2.4 L for 1 h. The consortia biomass treatment produces hydrolysates with high antioxidant capacity, mainly from the consortium C1, constituted by *C. vulgaris* and *A. platensis*, which can be attributed to the production of bioactive peptides due to enzymatic action, and also to the release of phenolic compounds during hydrolysis. Moreover, the in silico hydrolysis of the proteins present in both biomasses confirmed the release of low molecular weight peptides composed mainly of hydrophobic amino acids, linked to their antioxidant activity. Overall, the bioactive capacity of the hydrolysates obtained from both microalgae-cyanobacteria consortia reveals the potential of these natural ingredients for the cosmetic, nutraceutical, and pharmaceutical industries, offering an innovative and sustainable solution for the development of products with antioxidant properties.

## Figures and Tables

**Table 1:** Protein, carbohydrate, lipid, moisture, and inorganic material content of microalgae-cyanobacteria consortia (C1 and C2) biomasses.

Sample	Protein (%)		Carbohydrates (%)	Lipids (%)	Moisture (%)	Inorganic Material (%)
	Kjeldahl *	BCA				
C1	50.90 ± 0.03	48.65 ± 3.21	19.35 ± 0.74	6.22 ± 1.37	5.16 ± 0.03	9.20 ± 0.11
C2	25.98 ± 0.47	23.92 ± 2.27	35.48 ± 0.81	2.71 ± 0.72	5.11 ± 0.11	37.07 ± 0.08

* Conversion factor: 5.95. BCA: bicinchoninic acid. Results are expressed as mean ± standard deviation.

**Table 2:** Protein content of supernatants (soluble proteins) obtained at different solubilization and precipitation pH values from microalgae-cyanobacteria consortia C1 and C2.

Sample	Solubilization pH	Precipitation pH	Protein (%)
C1	10	5	30.59 ± 2.60 ^d^
		4	22.45 ± 1.48 ^c^
		3	12.66 ± 1.00 ^a^
	12	5	23.31 ± 1.83 ^c^
		4	17.28 ± 1.25 ^b^
		3	11.30 ± 0.66 ^a^
C2	10	5	9.16 ± 0.60 ^A^
		4	9.84 ± 0.59 ^B^
		3	9.99 ± 0.57 ^B^
	12	5	10.71 ± 0.57 ^C^
		4	8.76 ± 0.55 ^A^
		3	8.75 ± 0.96 ^A^

Results are expressed as mean ± standard deviation. ^a–d; A–C^: different letters indicate significant differences (*p* < 0.05); lowercase letters for C1 and uppercase letters for C2.

**Table 3:** Total protein content, total phenolic compounds, and chlorophylls a and b determined in the supernatants obtained from the Alcalase 2.4 L hydrolysates of microalgae-cyanobacteria consortia C1 and C2 at different incubation times.

Sample	Hydrolysis Time (h)	Protein (%)	Total Phenolic Compounds (mg GAE/g)	Chlorophyll a (mg/g)	Chlorophyll b (mg/g)
C1	0	23.46 ± 2.18 ^c^	14.39 ± 0.16 ^a^	1.15 ± 0.10 ^b^	2.31 ± 0.20 ^b^
	1	20.46 ± 1.59 ^b^	16.17 ± 1.41 ^b^	1.07 ± 0.06 ^b^	2.31 ± 0.19 ^b^
	3	17.14 ± 1.53 ^a^	16.31 ± 0.48 ^b^	0.85 ± 0.07 ^a^	1.73 ± 0.19 ^a^
	6	17.23 ± 1.65 ^a^	15.98 ± 0.72 ^b^	0.88 ± 0.05 ^a^	1.99 ± 0.20 ^ab^
C2	0	7.37 ± 0.54 ^A^	3.88 ± 0.15 ^A^	0.85 ± 0.08 ^B^	1.52 ± 0.13 ^C^
	1	9.76 ± 0.71 ^B^	6.25 ± 0.48 ^B^	0.85 ± 0.05 ^B^	1.23 ± 0.13 ^B^
	3	7.14 ± 0.75 ^A^	6.16 ± 0.45 ^B^	0.79 ± 0.06 ^B^	1.08 ± 0.12 ^AB^
	6	7.85 ± 0.69 ^A^	6.18 ± 0.46 ^B^	0.72 ± 0.04 ^A^	0.97 ± 0.10 ^A^

GAE: Gallic acid equivalents. Results are expressed as mean ± standard deviation. ^a–c; A–C^: different letters indicate significant differences over time (*p* < 0.05); lowercase letters for C1 and uppercase letters for C2.

**Table 4:** Selected peptide sequences released from consortia C1 and C2 biomasses after in silico hydrolysis with Alcalase which were predicted to be antioxidant by AnOxPP, AnOxPePred, and BIOPEP-UWM databases.

Biomass	Peptide	Antioxidant Reported Peptide	Source	Other Reported Bioactivity (Sequence and Activity)	References
C1/C2	GW	GW	Synthetic peptide	GW (antihypertensive, antidiabetic, peptidase inhibition)	[58,59,60,61]
C1/C2	PW	PW	Buckwheat	PW (antidiabetic)	[62]
C1/C2	GGW	-	-	GGW (anti-inflammatory)	[63]
C1	PGW	-	-	ASQSIWLPGWL (antihypertensive)	[64]
C1	PPY	RPDFDLEPPY	*Limanda aspera*	-	[65]
C1	PPW	FPPWVL	*A. platensis*	-	[66]
		FPPWF	*A. platensis*	-	[66]
C1	PHF	PHF	Synthetic peptide	-	[67]
C1	PHW	PHW	Synthetic peptide	-	[67]
C2	GGY	-	-	GGY (antihypertensive)	[68]
C2	GPW	AFDEGPWPK	Rice bran	-	[69]
C2	HGF	VKRRGQDCIHGFCSD	*L. fragilis*	-	[70]
C2	GGGW	-	-	GGGW (antidiabetic)	[71]

## Data Availability

The original contributions presented in this study are included in the article/Supplementary Materials. Further inquiries can be directed to the corresponding author. The mass spectrometry proteomics data have been deposited in the ProteomeXchange Consortium via the PRIDE partner repository with the dataset identifier PXD077201 for C1 and PXD077149 for C2.

## Supplementary Materials

The following supporting information can be downloaded at: https://www.mdpi.com/article/10.3390/md24050184/s1, Table S1: Identified proteins from consortium 1 (C1) composed by microalgae and cyanobacteria species *Chlorella vulgaris* and *Arthrospira platensis*. Table S2: Identified proteins from consortium 2 (C2) composed by cyanobacteria and microalgae species *Kamptonema* sp., *Nannochloropsis oculata*, *Tetraselmis suecica*, and *Chlorella vulgaris*. Table S3: Physicochemical characterization using ToxinPrep software of the estimated antioxidant peptide sequences released from consortia C1 and C2 following in silico AnOxPP and AnOxPePred database analysis.

## References

[1] Zhou L., Li K., Duan X., Hill D., Barrow C., Dunshea F., Martin G., Suleria H. (2022). Bioactive compounds in microalgae and their potential health benefits. Food Biosci..

[2] Rojas V., Rivas L., Cárdenas C., Guzmán F. (2020). Cyanobacteria and eukaryotic microalgae as emerging sources of antibacterial peptides. Molecules.

[3] Barkia I., Saari N., Manning S.R. (2019). Microalgae for high-value products towards human health and nutrition. Mar. Drugs..

[4] Águila-Carricondo P., de la Roche Cadavid J.P., Galán P.L., Bautista L.F., Vicente G. (2023). New green biorefineries from cyanobacterial-microalgal consortia: Production of chlorophyll-rich extracts for the cosmetic industry and sustainable biogas. J. Clean. Prod..

[5] Torres-Tiji Y., Fields F.J., Mayfield S.P. (2020). Microalgae as a future food source. Biotechnol. Adv..

[6] Ampofo J., Abbey L. (2022). Microalgae: Bioactive composition, health benefits, safety and prospects as potential high-value ingredients for the functional food industry. Foods.

[7] Caporgno M.P., Mathys A. (2018). Trends in microalgae incorporation into innovative food products with potential health benefits. Front. Nutr..

[8] Zheng H., Yin J., Gao Z., Huang H., Ji X., Dou C. (2011). Disruption of Chlorella vulgaris cells for the release of biodiesel-producing lipids: A comparison of grinding, ultrasonication, bead milling, enzymatic lysis, and microwaves. Appl. Biochem. Biotechnol..

[9] Benelhadj S., Gharsallaoui A., Degraeve P., Attia H., Ghorbel D. (2016). Effect of pH on the functional properties of Arthrospira (Spirulina) platensis protein isolate. Food Chem..

[10] Soto-Sierra L., Stoykova P., Nikolov Z.L. (2018). Extraction and fractionation of microalgae-based protein products. Algal Res..

[11] Tejano L.A., Peralta J.P., Yap E.E.S., Chang Y.W. (2019). Bioactivities of enzymatic protein hydrolysates derived from Chlorella sorokiniana. Food Sci. Nutr..

[12] Mahdieh G., Fazilati M., Izadi M., Pilehvarian A., Nazem H. (2020). Investigation of ACE inhibitory effect and antioxidant activity of peptide extracted from Spirulina platensis. Chem. Methodol..

[13] Chen Y., Chen J., Zheng Y., Yu H., Zhao J., Chen J., Zhu J. (2021). Dunaliella salina-derived peptide protects from bone loss: Isolation, purification and identification. LWT.

[14] Zhang Y., Jiang W., Hao X., Tan J., Wang W., Yu M., Zhang G., Zhang Y. (2021). Preparation of the enzymatic hydrolysates from Chlorella vulgaris protein and assessment of their antioxidant potential using Caenorhabditis elegans. Mol. Biotechnol..

[15] Cruz-Casas D.E., Aguilar C.N., Ascacio-Valdés J.A., Rodríguez-Herrera R., Chávez-González M.L., Flores-Gallegos A.C. (2021). Enzymatic hydrolysis and microbial fermentation: The most favorable biotechnological methods for the release of bioactive peptides. Food Chem..

[16] Akbarian M., Khani A., Eghbalpour S., Uversky V.N. (2022). Bioactive peptides: Synthesis, sources, applications, and proposed mechanisms of action. Int. J. Mol. Sci..

[17] Ashraf A., Guo Y., Yang T., Ud Din A.S., Ahmad K., Li W., Hou H. (2025). Microalgae-derived peptides: Exploring bioactivities and functional food innovations. J. Agric. Food Chem..

[18] Li Y., Lammi C., Boschin G., Arnoldi A., Aiello G. (2019). Recent advances in microalgae peptides: Cardiovascular health benefits and analysis. J. Agric. Food Chem..

[19] Fernando R., Sun X., Rupasinghe H.P.V. (2024). Production of bioactive peptides from microalgae and their biological properties related to cardiovascular disease. Macromol.

[20] Souza A.T.V., Souza K.M.S., Amorim A.P., Bezerra R.P., Porto A.L.F. (2024). Methods to protein and peptide extraction from microalgae: A systematic review. An. Acad. Bras. Cienc..

[21] Pekkoh J., Ruangrit K., Pumas C., Duangjan K., Chaipoot S., Phongphisutthinant R., Jeerapan I., Sawangrat K., Pathom-aree W., Srinuanpan S. (2023). Transforming microalgal Chlorella biomass into cosmetically and nutraceutically protein hydrolysates using high-efficiency enzymatic hydrolysis approach. Biomass Convers. Biorefin..

[22] Skjånes K., Aesoy R., Herfindal L., Skomedal H. (2021). Bioactive peptides from microalgae: Focus on anti-cancer and immunomodulating activity. Physiol. Plant..

[23] Lucakova S., Branyikova I., Hayes M. (2022). Microalgal proteins and bioactives for food, feed, and other applications. Appl. Sci..

[24] Cunha S.A., Coscueta E.R., Nova P., Silva J.L., Pintado M.M. (2022). Bioactive hydrolysates from Chlorella vulgaris: Optimal process and bioactive properties. Molecules.

[25] Böcker L., Bertsch P., Wenner D., Teixeira S., Bergfreund J., Eder S., Fischer P., Mathys A. (2021). Effect of Arthrospira platensis microalgae protein purification on emulsification mechanism and efficiency. J. Colloid. Interface Sci..

[26] Semerci A.B., Tekbaba A.G., Sevindik T.O. (2025). The effect of different culture mediums on the morphological characters, growth parameters, chemical contents, and biological activities of Kamptonema formosum (Bory ex Gomont) Strunecký, Komárek & J. Smarda. Braz. J. Microbiol..

[27] Zanella L., Vianello F. (2020). Microalgae of the genus Nannochloropsis: Chemical composition and functional implications for human nutrition. J. Funct. Foods..

[28] Hayes M., Mora L., Lucakova S. (2022). Identification of bioactive peptides from Nannochloropsis oculata using a combination of enzymatic treatment, in silico analysis and chemical synthesis. Biomolecules.

[29] Tulli F., Chini Zittelli G., Giorgi G., Poli B.M., Tibaldi E., Tredici M.R. (2012). Effect of the inclusion of dried Tetraselmis suecica on growth, feed utilization, and fillet composition of European sea bass juveniles fed organic diets. J. Aquat. Food Prod. Technol..

[30] Venckus P., Cicchi B., Chini Zittelli G. (2021). Effects of medium salinity on growth and biochemical composition of the green microalga Tetraselmis suecica. J. Appl. Phycol..

[31] Maltsev Y., Kulikovskiy M., Maltseva S. (2023). Nitrogen and phosphorus stress as a tool to induce lipid production in microalgae. Microb. Cell Fact..

[32] Wang Y., Tibbetts S.M., McGinn P.J. (2021). Microalgae as sources of high-quality protein for human food and protein supplements. Foods.

[33] Han Y., Ma B., Zhang K. (2005). SPIDER: Software for protein identification from sequence tags with de novo sequencing error. J. Bioinform. Comput. Biol..

[34] Rashid M.H.U., Yi E.K.J., Amin N.D.M., Ismail M.N. (2024). An empirical analysis of Sacha Inchi (Plantae: Plukenetia volubilis L.) seed proteins and their applications in the food and biopharmaceutical industries. Appl. Biochem. Biotechnol..

[35] Guadalupi L.S., Bianco M., Cataldi T.R.I., Ravnsborg T., Jensen O.N., Calvano C.D. (2025). Ultrasound-assisted protein extraction for deep proteome analysis of Spirulina and Chlorella microalgae. LWT.

[36] Bianco M., Ventura G., Calvano C.D., Losito I., Cataldi T.R.I. (2022). A new paradigm to search for allergenic proteins in novel foods by integrating proteomics analysis and in silico sequence homology prediction: Focus on spirulina and Chlorella microalgae. Talanta.

[37] Ismaiel M.M.S., Piercey-Normore M.D., Rampitsch C. (2018). Proteomic analyses of the cyanobacterium Arthrospira (Spirulina) platensis under iron and salinity stress. Environ. Exp. Bot..

[38] Ji C., Han J., Zhang J., Hu J., Fu Y., Qi H., Sun Y., Yu C. (2018). Omics-prediction of bioactive peptides from the edible cyanobacterium Arthrospira platensis proteome. J. Sci. Food Agric..

[39] Shishido T.K., Delbaje E., Wahlsten M., Vuori I., Jokela J., Gugger M., Fiore M.F., Fewer D.P. (2023). A cylindrospermopsin-producing cyanobacterium isolated from a microbial mat in the Baltic Sea. Toxicon.

[40] Tran N.A.T., Padula M.P., Evenhuis C.R., Commault A.S., Ralph P.J., Tamburic B. (2016). Proteomic and biophysical analyses reveal a metabolic shift in nitrogen deprived Nannochloropsis oculata. Algal Res..

[41] Hamzelou S., Belobrajdic D., Juhász A., Brook H., Bose U., Colgrave M.L., Broadbent J.A. (2023). Nutrition, allergenicity and physicochemical qualities of food-grade protein extracts from Nannochloropsis oculata. Food Chem..

[42] Guzmán F., Wong G., Román T., Cárdenas C., Alvárez C., Schmitt P., Albericio F., Rojas V. (2019). Identification of antimicrobial peptides from the microalgae Tetraselmis suecica (Kylin) Butcher and bactericidal activity improvement. Mar. Drugs..

[43] Lauritano C., De Luca D., Amoroso M., Benfatto S., Maestri S., Racioppi C., Esposito F., Lanora A. (2019). New molecular insights on the response of the green alga Tetraselmis suecica to nitrogen starvation. Sci. Rep..

[44] Akbarbaglu Z., Ayaseh A., Ghanbarzadeh B., Sarabandi K., Kharazmi M.S., Jafari S.M. (2023). Chemical structure and bio-functional properties of Arthrospira platensis peptides produced by ultrasonication-enzymolysis: Their emulsification capabilities. Process Biochem..

[45] Shishavan M.M., Mirdamadi S., Ofoghi H. (2019). Antioxidant activity of alcalase hydrolysates of Spirulina proteins. Adv. Res. Microb. Metab. Technol..

[46] Pekkoh J., Kamngoen A., Wichaphian A., Zin M.T., Chaipoot S., Yakul K., Pathomaree W., Maneechote W., Cheirsilp B., Khoo K.S. (2025). Production of ACE inhibitory peptides via ultrasonic-assisted enzymatic hydrolysis of microalgal Chlorella protein: Process improvement, fractionation, identification, and in silico structure-activity relationship. Future Foods.

[47] Md Saleh N.I., Ghani W.A.W.A., Harun M.R., Kamal S.M.M. (2019). Optimization of enzymatic hydrolysis for the production of antioxidative peptide from Nannochloropsis gaditana using response surface methodology. Sci. Technol..

[48] Quan T.H., Benjakul S., Sae-leaw T., Balange A.K., Maqsood S. (2019). Protein–polyphenol conjugates: Antioxidant property, functionalities and their applications. Trends Food Sci. Technol..

[49] Wang P., Grimm B. (2015). Organization of chlorophyll biosynthesis and insertion of chlorophyll into the chlorophyll-binding proteins in chloroplasts. Photosynth. Res..

[50] Solymosi K., Mysliwa-Kurdziel B. (2017). Chlorophylls and their derivatives used in food industry and medicine. Mini Rev. Med. Chem..

[51] Agregán R., Munekata P.E.S., Franco D., Carballo J., Barba F.J., Lorenzo J.M. (2018). Antioxidant potential of extracts obtained from macro- (Ascophyllum nodosum, Fucus vesiculosus and Bifurcaria bifurcata) and microalgae (Chlorella vulgaris and Spirulina platensis) assisted by ultrasound. Medicines.

[52] Alzahrani M.A.J., Perera C.O., Hemar Y. (2018). Production of bioactive proteins and peptides from the diatom Nitzschia laevis and comparison of their in vitro antioxidant activities with those from Spirulina platensis and Chlorella vulgaris. Int. J. Food Sci. Technol..

[53] Otero C., Verdasco-Martín C.M. (2023). Preparation and characterization of a multicomponent Arthrospira platensis biomass hydrolysate with superior anti-hypertensive, anti-hyperlipidemic and antioxidant activities via selective proteolysis. Mar. Drugs..

[54] Tacias-Pascacio V.G., Morellon-Sterling R., Siar E.H., Tavano O., Berenguer-Murcia Á., Fernandez-Lafuente R. (2020). Use of Alcalase in the production of bioactive peptides: A review. Int. J. Biol. Macromol..

[55] Zou T.B., He T.P., Li H.B., Tang H.W., Xia E.Q. (2016). The structure-activity relationship of the antioxidant peptides from natural proteins. Molecules.

[56] Xu B., Dong Q., Yu C., Chen H., Zhao Y., Zhang B., Yu P., Chen M. (2024). Advances in research on the activity evaluation, mechanism and structure-activity relationships of natural antioxidant peptides. Antioxidants.

[57] Jeong S., Jung J.H., Jung K.W., Ryu S., Lim S. (2023). From microbes to molecules: A review of microbial-driven antioxidant peptide generation. World J. Microbiol. Biotechnol..

[58] Du Z., Li Y. (2022). Computer-aided approaches for screening antioxidative dipeptides and application to sorghum proteins. ACS Food Sci. Technol..

[59] Cheung H.S., Wang F.L., Ondetti M.A., Sabo E.F., Cushman D.W. (1980). Binding of peptide substrates and inhibitors of angiotensin-converting enzyme. Importance of the COOH-terminal dipeptide sequence. J. Biol. Chem..

[60] Lan V.T., Ito K., Ohno M., Motoyama T., Ito S., Kawarasaki Y. (2015). Analyzing a dipeptide library to identify human dipeptidyl peptidase IV inhibitor. Food Chem..

[61] Ganellin C.R., Bishop P.B., Bambal R.B., Chan S.M., Law J.K., Marabout B., Luthra P.M., Moore A.N., Peschard O., Bourgeat P. (2000). Inhibitors of tripeptidyl peptidase II. 2. Generation of the first novel lead inhibitor of cholecystokinin-8-inactivating peptidase: A strategy for the de-sign of peptidase inhibitors. J. Med. Chem..

[62] Ma Y., Xiong Y.L., Zhai J., Zhu H., Dziubla T. (2010). Fractionation and evaluation of radical-scavenging peptides from in vitro digests of buckwheat protein. Food Chem..

[63] Wang S., Zheng L., Zhao T., Zhang Q., Liu Y., Sun B., Su G., Zhao M. (2020). Inhibitory effects of walnut (Juglans regia) peptides on neuroinflammation and oxidative stress in lipopolysaccharide-induced cognitive impairment mice. J. Agric. Food Chem..

[64] Montone C.M., Zenezini Chiozzi R., Marchetti N., Cerrato A., Antonelli M., Capriotti A.L., Cavaliere C., Piovesana S., Laganà A. (2019). Peptidomic approach for the identification of peptides with potential antioxidant and anti-hyperthensive effects derived from asparagus by-products. Molecules.

[65] Jun S.Y., Park P.J., Jung W.K., Kim S.K. (2004). Purification and characterization of an antioxidative peptide from enzymatic hydrolysate of yellowfin sole (Limanda aspera) frame protein. Eur. Food Res. Technol..

[66] Zhao C., Li F., Yan S., Zhu L., Ma S., Zhang T., Zhang N., Fang H., Du G. (2025). Identification and activity assay in vivo and in vitro of novel antioxidant and anti-aging peptides from C-phycocyanin of Limnospira platensis. Algal Res..

[67] Saito K., Jin D.H., Ogawa T., Muramoto K., Hatakeyama E., Yasuhara T., Nokihara K. (2003). Antioxidative properties of tripeptide libraries prepared by the combinatorial chemistry. J. Agric. Food Chem..

[68] Saito Y., Wanezaki K., Kawato A., Imayasu S. (1994). Structure and activity of angiotensin I converting enzyme inhibitory peptides from sake and sake lees. Biosci. Biotechnol. Biochem..

[69] Ren L.K., Fan J., Yang Y., Liu X.F., Wang B., Bian X., Wang D.F., Xu Y., Liu B.X., Zhu P.Y. (2023). Identification, in silico selection, and mechanism study of novel antioxidant peptides derived from the rice bran protein hydrolysates. Food Chem..

[70] Yu H., Qiao X., Gao J., Wang C., Cai S., Feng L., Wang H., Wang Y.P. (2015). Identification and characterization of novel antioxidant peptides involved in redox homeostasis of frog, Limnonectes fragilis. Protein Pept. Lett..

[71] Carrera-Alvarado G., Toldrá F., Mora L. (2022). DPP-IV inhibitory peptides GPF, IGL, and GGGW obtained from chicken blood hydrolysates. Int. J. Mol. Sci..

[72] De la Roche Cadavid J.P., Galán Gómez P.L. (2017). Método de cultivo, sistema de cultivo y biomasa de consorcios ad-hoc de microalgas y cianobacterias en biofilm con fines industriales.

[73] Bradstreet R.B. (1954). Kjeldahl method for organic nitrogen. Anal. Chem..

[74] Waghmare A.G., Salve M.K., LeBlanc J.G., Arya S.S. (2016). Concentration and characterization of microalgae proteins from Chlorella pyrenoidosa. Bioresour. Bioprocess..

[75] Figueiredo A.R.P., da Costa E., Silva J., Domingues M.R., Domingues P. (2019). The effects of different extraction methods of lipids from Nannochloropsis oceanica on the contents of omega-3 fatty acids. Algal Res..

[76] Albalasmeh A.A., Berhe A.A., Ghezzehei T.A. (2013). A new method for rapid determination of carbohydrate and total carbon concentrations using UV spectrophotometry. Carbohydr. Polym..

[77] Harris G.K., Marshall M.R. (2017). Food Analysis.

[78] Sanchiz Á., Morato E., Rastrojo A., Camacho E., González-de la Fuente S.G., Marina A., Aguado B., Requena J.M. (2020). The experimental proteome of Leishmania infantum promastigote and its usefulness for improving gene annotations. Genes.

[79] Shevchenko A., Wilm M., Vorm O., Mann M. (1982). Techniques in Protein Chemistry V.

[80] Alonso R., Pisa D., Marina A.I., Morato E., Rábano A., Rodal I., Carrasco L. (2015). Evidence for fungal infection in cerebrospinal fluid and brain tissue from patients with amyotrophic lateral sclerosis. Int. J. Biol. Sci..

[81] Tran N.H., Qiao R., Xin L., Chen X., Liu C., Zhang X., Shan B., Ghodsi A., Li M. (2019). Deep learning enables de novo peptide sequencing from data-independent-acquisition mass spectrometry. Nat. Methods..

[82] Tran N.H., Zhang X., Xin L., Shan B., Li M. (2017). De novo peptide sequencing by deep learning. Proc. Natl. Acad. Sci. USA.

[83] Tran N.H., Rahman M.Z., He L., Xin L., Shan B., Li M. (2016). Complete de novo assembly of monoclonal anti-body sequences. Sci. Rep..

[84] Perez-Riverol Y., Bandla C., Kundu D.J., Kamatchinathan S., Bai J., Hewapathirana S., John N.S., Prakash A., Walzer M., Wang S. (2025). The PRIDE database at 20 years: 2025 update. Nucleic Acids Res..

[85] Nielsen P.M., Petersen D., Dambmann C. (2001). Improved method for determining food protein degree of hydrolysis. J. Food Sci..

[86] Lichtenthaler H.K., Buschmann C. (2001). Chlorophylls and carotenoids: Measurement and characterization by UV-VIS spectroscopy. Curr. Protoc. Food Anal. Chem..

[87] Maadane A., Merghoub N., Ainane T., El Arroussi H., Benhima R., Amzazi S., Bakri Y., Wahby I. (2015). Antioxidant activity of some Moroccan marine microalgae: Pufa profiles, carotenoids and phenolic content. J. Biotechnol..

[88] Singleton V.L., Orthofer R., Lamuela-Raventós R.M. (1999). Analysis of total phenols and other oxidation substrates and antioxidants by means of Folin-Ciocalteu reagent. Methods Enzymol..

[89] Hernández-Ledesma B., Dávalos A., Bartolomé B., Amigo L. (2005). Preparation of antioxidant enzymatic hydrolysates from alpha-lactalbumin and beta-lactoglobulin. Identification of active peptides by HPLC-MS/MS. J. Agric. Food Chem..

[90] Re R., Pellegrini N., Proteggente A., Pannala A., Yang M., Rice-Evans C. (1999). Antioxidant activity applying an improved ABTS radical cation decolorization assay. Free Radic. Biol. Med..

[91] Maillet N. (2019). Rapid Peptides Generator: Fast and efficient in silico protein digestion. NAR Genom. Bioinform..

[92] Mooney C., Haslam N.J., Pollastri G., Shields D.C. (2012). Towards the improved discovery and design of functional peptides: Common features of diverse classes permit generalized prediction of bioactivity. PLoS ONE.

[93] Qin D., Jiao L., Wang R., Zhao Y., Hao Y., Liang G. (2023). Prediction of antioxidant peptides using a quantitative structure−activity relationship predictor (AnOxPP) based on bidirectional long short-term memory neural network and interpretable amino acid descriptors. Comput. Biol. Med..

[94] Olsen T.H., Yesiltas B., Marin F.I., Pertseva M., García-Moreno P.J., Gregersen S., Overgaard M.T., Jacobsen C., Lund O., Hansen E.B. (2020). AnOxPePred: Using deep learning for the prediction of antioxidative properties of peptides. Sci. Rep..

[95] Gupta S., Kapoor P., Chaudhary K., Gautam A., Kumar R., Raghava G.P.S., Open source drug discovery consortium (2013). In silico approach for predicting toxicity of peptides and proteins. PLoS ONE.

[96] Minkiewicz P., Iwaniak A., Darewicz M. (2019). BIOPEP-UWM database of bioactive peptides: Current opportunities. Int. J. Mol. Sci..

