# Supplementary material for: Enhancing the Recovery of Antioxidant Compounds from Microalgae-Cyanobacteria Consortia Through Alcalase Hydrolysis: A Focus on Bioactive Peptides
Source: Mar Drugs. 2026 May 20;24(5):184. doi: 10.3390/md24050184 (PMC13208806; doi:10.3390/md24050184)
Supplement: Supplementary file 1 [file marinedrugs-24-00184-s001.zip › Supplementary table S3_Pardo.pdf]

Supplementary Table S3. Physicochemical characterization using ToxinPrep software of the estimated antioxidant peptide sequences released from microalga-cyanobacteria consortia C1 and C2 following the in silico AnOxPP and AnOxPePred databases analysis.

Biomass	Peptide	Peptide Ranker	Numer of residues	MW (Da)	pI	Toxicity	SVM
C1/C2	GW	0.993164	2	261.30	5.88	Non-Toxin	-0.80
C1/C2	PW	0.992911	2	301.36	5.88	Non-Toxin	-0.79
C1/C2	GGW	0.986969	3	318.37	5.88	Non-Toxin	-0.77
C1/C2	HPM	0.824607	3	383.50	7.10	Non-Toxin	-0.77
C1/C2	GPGGL	0.884327	5	399.52	5.88	Non-Toxin	-0.54
C1/C2	SPGW	0.960144	4	445.52	5.88	Non-Toxin	-0.85
C1/C2	ICPY	0.814442	4	494.65	5.84	Non-Toxin	-0.68
C1/C2	VPGHF	0.823146	5	555.70	7.10	Non-Toxin	-0.97
C1/C2	PPPPSPF	0.970562	7	737.93	5.88	Non-Toxin	-0.53
C1/C2	GPPPPSPF	0.967732	8	795.00	5.88	Non-Toxin	-0.30
C1	PGY	0.825075	3	335.39	5.88	Non-Toxin	-0.80
C1	GCY	0.901043	3	341.41	5.84	Non-Toxin	-0.46
C1	PGW	0.987911	3	358.43	5.88	Non-Toxin	-0.81
C1	PPY	0.868141	3	375.45	5.88	Non-Toxin	-0.75
C1	PGGY	0.841936	4	392.46	5.88	Non-Toxin	-0.79
C1	PPW	0.989425	3	398.49	5.88	Non-Toxin	-0.81
C1	PHF	0.938016	3	399.48	7.10	Non-Toxin	-0.82
C1	GNGW	0.929791	4	432.49	5.88	Non-Toxin	-0.84
C1	PHW	0.955428	3	438.52	7.10	Non-Toxin	-0.80
C1	PGHF	0.950238	4	456.55	7.10	Non-Toxin	-0.91
C1	IPGW	0.95955	4	471.61	5.88	Non-Toxin	-0.71
C1	GPPPL	0.944984	5	479.64	5.88	Non-Toxin	-0.25
C1	GPRW	0.974974	4	514.63	10.11	Non-Toxin	-0.72
C1	HHPF	0.886714	4	536.64	7.26	Non-Toxin	-0.77
C1	RPPW	0.970586	4	554.69	10.11	Non-Toxin	-0.72
C2	GGY	0.814993	3	295.33	5.88	Non-Toxin	-0.74
C2	GPW	0.988841	3	358.43	5.88	Non-Toxin	-0.75
C2	HGF	0.938552	3	359.42	7.10	Non-Toxin	-0.82
C2	GGGW	0.975781	4	375.44	5.88	Non-Toxin	-0.78
C2	CPY	0.893457	3	381.47	5.84	Non-Toxin	-0.79
C2	HGW	0.948028	3	398.46	7.10	Non-Toxin	-0.79
C2	PGGW	0.979581	4	415.50	5.88	Non-Toxin	-0.82
C2	PPGY	0.895074	4	432.52	5.88	Non-Toxin	-0.61
C2	GPPPA	0.85984	5	437.55	5.88	Non-Toxin	-0.17
C2	HPW	0.957615	3	438.52	7.10	Non-Toxin	-0.72
C2	HPGF	0.960589	4	456.55	7.10	Non-Toxin	-0.68
C2	RPW	0.974653	3	457.56	10.11	Non-Toxin	-0.75
C2	VPGW	0.911065	4	457.58	5.88	Non-Toxin	-0.71
C2	HPPL	0.844231	4	462.60	7.10	Non-Toxin	-0.65
C2	PPPPA	0.876745	5	477.61	5.88	Non-Toxin	-0.32
C2	PGPPL	0.927501	5	479.64	5.88	Non-Toxin	-0.35

C2	PGRY	0.819568	4	491.59	9.10	Non-Toxin	-0.70
C2	VPPW	0.931491	4	497.64	5.88	Non-Toxin	-0.83
C2	GHIW	0.849315	4	511.64	7.10	Non-Toxin	-0.82
C2	PPPPL	0.956137	5	519.70	5.88	Non-Toxin	-0.33
C2	PPPPQ	0.84261	5	534.67	5.88	Non-Toxin	-0.32
C2	PPPPK	0.850923	5	534.71	9.11	Non-Toxin	-0.33
C2	HRGW	0.886633	4	554.66	10.11	Non-Toxin	-0.68
C2	HPHW	0.905596	4	575.68	7.26	Non-Toxin	-0.69
C2	PPPRF	0.981797	5	612.78	10.11	Non-Toxin	-0.63
C2	NHPCF	0.951661	5	616.75	7.06	Non-Toxin	-0.41
C2	PPPPPL	0.966059	6	616.83	5.88	Non-Toxin	-0.29
C2	IRHPF	0.841961	5	668.86	10.11	Non-Toxin	-0.47
C2	PPPPPGQ	0.883546	7	688.87	5.88	Toxin	0.08
C2	NPPNPPL	0.885629	7	747.94	5.88	Non-Toxin	-0.40
C2	GPGHGGPGL	0.815775	9	747.95	7.10	Non-Toxin	-0.64
C2	DVTPPPF	0.807767	7	771.95	3.80	Non-Toxin	-0.70
C2	PPPPRPE	0.810411	7	788.98	6.36	Non-Toxin	-0.51
C2	PPVCTPPPPL	0.90219	10	1017.38	5.85	Non-Toxin	-0.49
